# Different genomic relationship matrices for single-step analysis using phenotypic, pedigree and genomic information

**DOI:** 10.1186/1297-9686-43-1

**Published:** 2011-01-05

**Authors:** Selma Forni, Ignacio Aguilar, Ignacy Misztal

**Affiliations:** 1 Genus Plc, Hendersonville, TN, USA; 2 Instituto Nacional de Investigación Agropecuaria, Las Brujas, Uruguay; 3 Department of Animal and Dairy Science, University of Georgia, Athens, GA, USA

## Abstract

**Background:**

The incorporation of genomic coefficients into the numerator relationship matrix allows estimation of breeding values using all phenotypic, pedigree and genomic information simultaneously. In such a single-step procedure, genomic and pedigree-based relationships have to be compatible. As there are many options to create genomic relationships, there is a question of which is optimal and what the effects of deviations from optimality are.

**Methods:**

Data of litter size (total number born per litter) for 338,346 sows were analyzed. Illumina PorcineSNP60 BeadChip genotypes were available for 1,989. Analyses were carried out with the complete data set and with a subset of genotyped animals and three generations pedigree (5,090 animals). A single-trait animal model was used to estimate variance components and breeding values. Genomic relationship matrices were constructed using allele frequencies equal to 0.5 (**G05**), equal to the average minor allele frequency (**GMF**), or equal to observed frequencies (**GOF**). A genomic matrix considering random ascertainment of allele frequencies was also used (GOF*). A normalized matrix (**GN**) was obtained to have average diagonal coefficients equal to 1. The genomic matrices were combined with the numerator relationship matrix creating **H **matrices.

**Results:**

In **G05 **and **GMF**, both diagonal and off-diagonal elements were on average greater than the pedigree-based coefficients. In **GOF **and GOF*, the average diagonal elements were smaller than pedigree-based coefficients. The mean of off-diagonal coefficients was zero in **GOF **and GOF*. Choices of **G **with average diagonal coefficients different from 1 led to greater estimates of additive variance in the smaller data set. The correlation between EBV and genomic EBV (n = 1,989) were: 0.79 using **G05**, 0.79 using **GMF**, 0.78 using **GOF**, 0.79 using GOF*, and 0.78 using **GN**. Accuracies calculated by inversion increased with all genomic matrices. The accuracies of genomic-assisted EBV were inflated in all cases except when **GN **was used.

**Conclusions:**

Parameter estimates may be biased if the genomic relationship coefficients are in a different scale than pedigree-based coefficients. A reasonable scaling may be obtained by using observed allele frequencies and re-scaling the genomic relationship matrix to obtain average diagonal elements of 1.

## Background

Traditional genetic evaluation of livestock combines only phenotypic data and probabilities that genes are identical by descent using the pedigree information. Genetic markers for many loci across the genome can be used to measure genetic similarity and may be more precise than pedigree information [1]. Genomic relationships can better estimate the proportion of chromosomes segments shared by individuals because high-density genotyping identifies genes identical in state that may be shared through common ancestors not recorded in the pedigree. A genomic relationship matrix (**G**) can be calculated by different methods [1,2].

As an entire population is unlikely to be genotyped in livestock species, Legarra et al. [3] and Misztal et al. [4] have proposed the integration of the numerator relationship matrix (**A**) and **G **into a single matrix (**H**). A BLUP evaluation using **H **called single-step genomic evaluation has been successfully applied in dairy cattle [5]. Besides the computation of **H**, no further modifications in the standard mixed model equations used in animal breeding have been needed [4].

The formula for **H **includes the expression **G **- **A**, which is the difference between genomic and pedigree-based relationships. If **G **is inflated, deflated or in some other way incompatible with **A**, the weighting of the pedigree and genomic information will be incorrect. Various **G **used in a genetic evaluation by Aguilar et al. [5] have resulted in different scaling and accuracies of EBV. Estimates of the additive variance using **G **may be much larger than those using **A **[6]. Different **G **can lead to different accuracies of EBV [5]. These differences could be due to an incorrect scaling of **G **relative to **A**.

The first objective of this study was to apply different genomic matrices to analyses of litter size in a swine population and evaluate the impact of those **G **on EBV and estimates of variance components. The second objective was to develop a strategy to create an optimal **G **that is easy to create and yields reasonably accurate EBV and estimates of the additive variance.

## Methods

### Data

Data of litter size (total number born per litter) for 338,346 sows, of which 1,919 were genotyped using the Illumina PorcineSNP60 BeadChip, were analyzed. Genotypes of their 70 sires were also available. Genotyped females were crosses of two pure lines derived from the same breeds, and they were born in a two-year span. After quality control procedures, 44,298 markers remained and were used to estimate genomic relationship coefficients. In the quality control analysis, SNP were excluded if: the minor allele frequency was smaller than 0.05, the marker mapped to the sex chromosomes, the chi-square statistics for Hardy-Weinberg equilibrium from males and females differed by more than 0.1, or more than 20% of animals had missing genotypes. Phenotypes were collected in genetic nucleus (pure lines) and commercial herds (line crosses) and the parental lines were included as fixed effects in the model to account for differences in the genetic backgrounds. All analyses were carried out with the complete data set and with a subset containing only genotyped females and three generations of pedigree (5,090 animals). Records were analyzed using an animal model. Fixed effects included parity order, age at farrowing (linear covariable), number of services, mating type (artificial insemination or natural service), contemporary group, sow line and sire line (parents of animals with phenotype). Contemporary groups were defined by season, year and farrowing farm. The numerator relationship matrix was obtained with pedigree information on 382,988 animals. Prediction error variances (PEV) were obtained by inversion of the coefficients matrix of the mixed model equations.

### Combined pedigree-genomic relationship matrix

In the animal model, the inverse of the numerator relationship matrix (**A**^-1^) was replaced by **H**^-1 ^that combines the pedigree and genomic information [5]:

(1) $${\text{H}}^{-1}={\text{A}}^{-1}+\left[\begin{array}{cc} 0 & 0\\ 0 & {\text{G}}^{-1}-{\text{A}}_{22}^{-1} \end{array}\right],$$

where **G**^-1 ^is the inverse of the genomic relationship matrix and ${\text{A}}_{22}^{-1}$ is the inverse of the pedigree-based relationship matrix for genotyped animals. Comparisons involved several genomic relationship matrices. First, **G **was obtained following VanRaden [1]:

(2) $$\text{G}=\frac{\left(\text{M}-\text{P}\right){\left(\text{M}-\text{P}\right)}^{\prime }}{2\sum_{\text{j}=1}^{\text{m}}p_{j}(1-p_{j})},$$

where **M **is an allele-sharing matrix with m columns (m = total number of markers) and n rows (n = total number of genotyped individuals), and **P **is a matrix containing the frequency of the second allele (p*_j_*), expressed as 2p*_j_*. M*_ij _*was 0 if the genotype of individual *i *for SNP *j *was homozygous 11, was 1 if heterozygous, or 2 if the genotype was homozygous 22. Frequencies should be those from the unselected base population, but this information was not available. Instead the frequencies used were: 0.5 for all markers (**G05**), the average minor allele frequency (**GMF**), and the observed allele frequency of each SNP (**GOF**). The last option assured that the average off-diagonal element was close to 0. For **GMF **only, the second allele was the one with smaller frequency.

A different matrix with observed frequencies (GOF*) was obtained by modification of the denominator as in Gianola et al. [7]:

(3) $$\text{GOF}*=\frac{\left(\text{M}-\text{P}\right){\left(\text{M}-\text{P}\right)}^{\prime }}{\left[{\left({\text{p}}_{0}-{\text{q}}_{0}\right)}^{2}+2\left(\frac{\sum_{\text{j}=1}^{\text{m}}{\text{p}}_{j}(1-{\text{p}}_{j})}{\text{m}}\right)\left(\frac{\alpha +\beta +2}{\alpha +\beta }\right)\right]\text{m}},$$

where p_0 _and q_0 _are expectations of allele frequencies following a Beta distribution with hyperparameters α and β. The values for the hyperparameters were the same as observed in the genotyped animals. A normalized matrix was obtained to have average diagonal coefficients equal to 1:

(4) GN=(M−P)(M−P)′{trace[(M−P)(M−P)′]}n.

The denominator should assure compatibility with A when either the average inbreeding is low or the number of generations low. Higher levels of inbreeding in the genotyped population can be accommodated by substituting "n" in the denominator of **GN **by the sum of (1 + F) across genotyped animals, where F are individual inbreeding coefficients derived from pedigree. Different from the numerator relationship matrix, values on the diagonal of **GN **can be smaller than 1. An average diagonal of 1 can also be obtained by multiplying (4) by a constant. A similar relationship matrix with sample variance of 1 was used by Kang et al. [8].

The genomic matrix is positive semidefinite but it can be singular if the number of loci is limited or two individuals have identical genotypes across all markers. It will be singular if the number of markers is smaller than the number of individuals genotyped. To avoid potential problems with inversion, **G **was calculated as **G **= w**Gr **+ (1 - w)**A**_22_, where w = 0.95 and **Gr **is a genomic matrix before weighting. Tests showed that the value of w was not critical. Aguilar et al. [5] reported negligible differences in EBV using w between 0.95 and 0.98. Christensen and Lund [9] suggested that w could be interpreted as the relative weight of the polygenic effect needed to explain the total additive variance, such as: $\text{w}=\sigma_{\text{a}}^{2}/\left(\sigma_{\text{g}}^{2}+\sigma_{\text{a}}^{2}\right)$, where $\sigma_{\text{g}}^{2}$ is the variance explained by the markers.

The joint distribution of breeding values of genotyped (**a**_1_) and non-genotyped animals (**a**_2_) is:

(5) $$\begin{array}{c} \left(\begin{array}{l} {\text{a}}_{1}\\ {\text{a}}_{2} \end{array}\right)\sim \\ \left(0,\left[\begin{array}{cc} {\text{A}}_{11}+{\text{A}}_{12}{\text{A}}_{22}^{-1}\left(\text{G}-{\text{A}}_{22}\right){\text{A}}_{22}^{-1}{\text{A}}_{21} & {\text{A}}_{12}{\text{A}}_{22}^{-1}\text{G}\\ \text{G}{\text{A}}_{22}^{-1}{\text{A}}_{12} & \text{G} \end{array}\right]\sigma_{a}^{2}\right), \end{array}$$

and the variances of the conditional posterior distributions are:

(6a) $$\begin{array}{l} \mathrm{var}\left({\text{a}}_{1}|{\text{a}}_{2},\sigma_{a}^{2},\sigma_{e}^{2},\text{y}\right)=\\ {\left[{\text{A}}_{11}+{\text{A}}_{12}{\text{A}}_{22}^{-1}\left(\text{G}-{\text{A}}_{22}\right){\text{A}}_{22}^{-1}{\text{A}}_{21}\right]}^{-1}\sigma_{a}^{2}, \end{array}$$

(6b) $$\mathrm{var}\left({\text{a}}_{2}|{\text{a}}_{1},\sigma_{\text{a}}^{2},\sigma_{\text{e}}^{2},\text{y}\right)={\text{G}}^{-1}\sigma_{\text{a}}^{2}.$$

The additive variance is on average the same for the entire population, and coefficients of **A **and **G **need to be compatible in scale. Variance components were estimated by restricted maximum likelihood (REML) using the EM algorithm [10].

## Results

### Pedigree-based and genomic relationship coefficients

**Figure 1:**
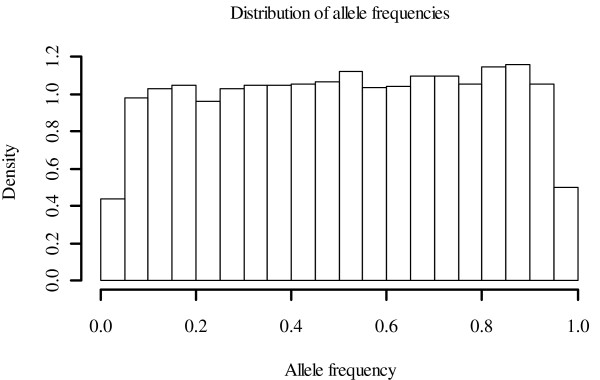
**Distribution of allele frequencies**. Observed frequencies of the second allele

Statistics of pedigree-based and genomic relationship coefficients for genotyped animals (**A**_22 _or **G**) are in Table 1. In **G05 **and **GMF**, the same allele frequency was used for all markers, and the average of both diagonal and off-diagonal elements was greater than the coefficients in **A**_22_. The average minor allele frequency was 0.26. The distribution of frequencies of the second allele was nearly flat (Figure 1). For **GOF **and GOF*, the average diagonal coefficients were smaller than the pedigree-based coefficients. The average off-diagonal coefficients were zero in both matrices, similar to **A**_22_. This allowed obtaining a matrix with average diagonal elements equal to 1 (**GN**) and average off-diagonal elements equal to zero. For all genomic matrices, diagonal coefficients had greater variance than the pedigree-based coefficients. Off-diagonal genomic coefficients had a greater variance only for **GOF **and **GN**. Greater variance was expected between the elements of **G **than **A **because genomic relationships reflect the actual fraction of genes shared whereas pedigree-based coefficients are predictions. Predictions have smaller variance than the variable predicted when the prediction error is not zero.

**Table 1:** Statistics of relationship coefficients estimated using pedigree and genomic information

Diagonal elements				
	Mean	Minimum	Maximum	Variance

**A**	1.000	1.000	1.075	0.00003
**G05**	1.253	1.178	1.462	0.00083
**GMF**	1.697	1.632	1.894	0.00073
**GOF**	0.936	0.837	1.228	0.00176
GOF*	0.505	0.436	0.663	0.00051
**GN**	1.002	0.895	1.314	0.00201

**Off-diagonal elements**				

	Mean	Minimum	Maximum	Variance

**A**	0.032	0.000	0.600	0.00172
**G05**	0.595	0.387	1.231	0.00160
**GMF**	1.022	0.822	1.654	0.00155
**GOF**	0.000	-0.198	1.000	0.00241
GOF*	0.000	-0.105	0.540	0.00070
**GN**	0.000	-0.212	1.070	0.00275

Relationships between genotyped animals (1,989 diagonal and 3,954,132 off-diagonal elements)

### Variance components

Estimates of variance components obtained with the full data set are in Table 2, and estimates from the subset are in Table 3. The differences observed in the complete data set were negligible, most likely because genomic relationships were a small fraction of all relationships. Compared to estimates obtained with **A**, most of the additive variance estimates using the genomic relationships in the smaller dataset were inflated. The inflation was approximately inversely proportional to the difference between the average diagonal and the off-diagonal elements of **G**. The highest inflation was with GOF*, for which this difference was only 0.51. The additive variance estimates were the same for **G05 **and **GMF **despite different averages but with similar differences between average diagonal and off-diagonal elements, 0.66 and 0.68, respectively. Estimates in the smaller data were similar using **A **and **GN**, which had very similar diagonal and off-diagonal element averages. Legarra et al. [3] have demonstrated that a normalized genomic matrix, as **GN **= **G**/trace(**G**), allows the same expectation of variance for breeding values of genotyped and non-genotyped animals. Assuming that a genomic relationship matrix standardized such as **GN **produces realistic estimates of additive variance, the use of genomic information resulted in smaller standard errors (0.30) than only pedigree information (0.44).

**Table 2:** Variance components estimates for litter size using pedigree and genomic relationship coefficients

	**Additive Variance (ste**^**1**^**)**	**Residual Variance (ste**^**1**^**)**
**A**	1.26 (± 0.03)	6.66 (± 0.02)
**G05**	1.28 (± 0.03)	6.65 (± 0.03)
**GMF**	1.28 (± 0.03)	6.65 (± 0.03)
**GOF**	1.27 (± 0.03)	6.65 (± 0.03)
GOF*	1.30 (± 0.03)	6.64 (± 0.03)
**GN**	1.27 (± 0.03)	6.65 (± 0.03)

Full data set (n = 338,346), ^1^ste = standard error

**Table 3:** Variance components estimates for litter size using pedigree and genomic relationship coefficients

	**Additive Variance (ste**^**1**^**)**	**Residual Variance (ste**^**1**^**)**
**A**	2.27 (± 0.52)	5.30 (± 0.44)
**G05**	3.43 (± 0.56)	5.25 (± 0.29)
**GMF**	3.43 (± 0.56)	5.25 (± 0.30)
**GOF**	2.41 (± 0.39)	5.29 (± 0.30)
GOF*	4.46 (± 0.73)	5.22 (± 0.30)
**GN**	2.25 (± 0.36)	5.30 (± 0.30)

Subset of genotyped animals (n = 1,919), ^1^ste = standard error

### Breeding values and accuracies

Estimates of breeding values for genotyped animals were on average similar regardless the choice of **G**. Table 4 presents correlations between breeding values obtained with different relationship matrices. Small differences were observed in the ranks obtained with different genomic matrices. However, these differences have direct implications on selection decisions and genetic progress. For instance, if 597 animals (top 30%) were selected using **GN**, 456 animals among the 597 would also be selected using **A**. For other genomic matrices, the number of animals selected in common with **GN **was: 567 for **G05**, 568 for **GMF**, 593 for **GOF**, and 554 for GOF*.

**Table 4:** Correlations between estimated breeding values using different relationship matrices

	A	G05	GMF	GOF	GOF*	GN
**A**		0.798	0.798	0.793	0.799	0.791
**G05**	0.891		1.000	0.995	0.997	0.993
**GMF**	0.891	1.000		0.995	0.997	0.994
**GOF**	0.891	0.997	0.997		0.989	0.999
GOF*	0.891	0.996	0.996	0.999		0.996
**GN**	0.888	0.998	0.998	0.997	0.986	

Genotyped females above diagonal (n = 1,919)

Genotyped males bellow diagonal (n = 70)

Correlations between pedigree-based EBV and EBV obtained using either **G05 **or **GN **were similar. When applied to dairy data, Aguilar et al. [5] have found substantially higher accuracies for **G **with allele frequencies equal to 0.5 than with either current or estimated base allele frequencies. When the allele frequency is p, the relative contribution to the diagonal of **G **is (2p)^2 ^for the first homozygote, (1-2p)^2 ^for a heterozygote, and (2-2p)^2 ^for the second homozygote. With p = 0.5, these contributions are 1, 0, and 1, respectively. When the allele frequencies are assumed different from 0.5, these contributions are different for each homozygote. For example, contributions with p = 0.2 would be 0.16 for the first homozygote, 0.36 for the heterozygote, and 2.56 for the second homozygote. Consequently, rare alleles contribute more to the variance than common alleles. It would be interesting to compare the results with a normalized matrix from **G05 **by multiplying and deducting a constant as in VanRaden [1]. However, in our experience such matrices were not positive definite. Subtracting of a constant from **G **might be helpful if this does not create a negative eigenvalue.

Statistics on computed breeding values with various relationship matrices are in Table 5. The means can be clustered in two groups, one for matrices based on the observed allele frequencies where the average off-diagonal is 0, and another for the remaining matrices. When the average off-diagonals were larger than zero, all genotyped animals were related with positive coefficients. The assumption that all animals are related may create biases especially when animals of interest have both phenotypes and genotypes. The exact impact of large off-diagonals is a topic for future research.

**Table 5:** Statistics of estimated breeding values using pedigree and genomic information

Genotyped females (n = 1,919)				
	Mean	Minimum	Maximum	Variance

**A**	0.359	-2.755	2.282	0.467
**G05**	0.372	-2.898	2.501	0.443
**GMF**	0.372	-2.904	2.505	0.444
**GOF**	0.165	-3.623	2.660	0.566
GOF*	0.165	-2.829	2.110	0.376
**GN**	0.165	-3.697	2.707	0.589

**Genotyped males (n = 70)**				

	Mean	Minimum	Maximum	Variance

**A**	0.159	-4.097	2.847	1.185
**G05**	0.135	-3.717	2.525	0.996
**GMF**	0.135	-3.722	2.524	0.998
**GOF**	-0.051	-4.428	2.509	1.160
GOF*	-0.040	-3.688	2.180	1.178
**GN**	-0.074	-4.502	2.522	0.905

Estimates of accuracy obtained using PEV with different genomic matrices are in Table 6. On average, the increase of accuracy from genomic information was for genotyped animals only. The increases were higher for females because of their lower initial accuracy. The accuracies varied depending on the genomic matrix used. Assuming that additive variance and accuracy estimates are most realistic with **GN**, the accuracies using non-normalized **G **were inflated. VanRaden et al. [11] have presented computed and realized genomic accuracies for a number of traits, and found the computed accuracies to be inflated.

**Table 6:** Average accuracy estimates for breeding values using pedigree and genomic relationship coefficients

	Full pedigree (n = 382,988)	Genotyped females (n = 1,919)	Genotyped sires (n = 70)
**A**	0.21	0.22	0.62
**G05**	0.21	0.37	0.63
**GMF**	0.21	0.49	0.64
**GOF**	0.21	0.30	0.63
GOF*	0.21	0.43	0.66
**GN**	0.21	0.28	0.63

## Discussion

Pedigrees may include many generations into the history of the population but must end eventually. In standard genetic evaluations, founder animals are the earliest generation recorded and the assumption is that they do not share genes from older ancestors. Relationship and inbreeding coefficients from later generations are estimated as deviations from the founders' relatedness. Genomic analysis typically reveals that founder animals actually share genes identical by descent, which shift relationship and inbreeding coefficients up or down. Genomic and pedigree-based matrices should be compatible in scale to be integrated. Ideally, genomic relationships should be estimated using the allele frequencies from the unselected base population. This information can be rarely extracted from historical data and approximations must to be used. Errors in the allele frequency estimates may result in biased relationships and consequently biased GEBVs, especially for young animals [5]. Yang et al. [12] have proposed a genomic relationship matrix that uses the genotyped animals as the base population. They have presented a slightly different formulation than used here for the diagonal elements of **G**. Using the genotyped population as base, **A **would have to be re-scaled according to **G **but allele frequencies in the base population would not have to be estimated.

Coefficients of **GN **had greater variance than the corresponding elements of **A**_22_. The variance was greater because individuals equally related in the pedigree have more or less alleles in common than expected. Genomic analysis achieved higher accuracies probably because genomic information improved prediction of the Mendelian sampling terms. More differentiation within families and reduction of co-selection of sibs are expected with genomic-assisted selection because Mendelian sampling can be better estimated. As a result, inbreeding across generations is expected to increase more slowly than it would increase with standard evaluations [13].

We considered only phenotypes of crossbred animals. The performance of crossbred animals is considered a different trait than the performance of purebred animals in routine evaluations of this population. Using a multitrait model, one can predict EBV for elite animals as parents at the nucleus and commercial level simultaneously. However, only additive inheritance is considered in this model and differences in allele frequencies between pure lines are ignored. Cantet and Fernando [14] have shown that ignoring segregation variance could lead to unbiased predictions that do not have the minimum variance. More suitable models should be used to account for heterosis when the objective is to rank crossbred animals [15,16].

Estimates of additive variance were sensitive to the choices of **G **when a greater part of the pedigree was genotyped. An entire genotyped population is rarely found in livestock species, and pedigree and genomic information have to be combined. Estimates of relationships are always relative to an arbitrary base population in which the average relationship is zero. Genomic and pedigree-based relationships must be relative to the same base to be combined in the **H **matrix. We chose to use the animals with unknown parents in **A **as the base, and we modified **G **accordingly. Because there were no changes in the genetic base, the same additive variance is expected when including the genomics coefficients. A practical solution to avoid inflation of the additive variance is to re-scale **G **to obtain average diagonal elements equal to 1, when off-diagonal elements are already on average zero. In the data set analyzed, average off-diagonal elements equal to zero were obtained using the observed allele frequencies.

Several studies have indicated accuracy gains with the inclusion of genomic information in genetic evaluations via marker regression or identical-by-descent matrices [11,17,18]. However, some experiences in the dairy industry, however, have indicated that actual improvement may differ from expected because of inflation of genomic breeding values and reliabilities [5,11]. Biases in genomic predictions can be related to incorrect weighting of polygenic and genomic components. The combined pedigree-genomic relationship matrix provides a natural way to weight both components for optimal predictions. In addition, a single-step genomic evaluation eliminates a number of assumptions and parameters required in multiple-step methods, and possibly delivers more accurate evaluations for young animals. The single-step procedure can be easily extended for multiple-traits analysis, and can handle large amounts of genomic information. Extensions to account for other distributions of marker effects, i.e., large QTL or major genes, are also possible [19,20]. Nevertheless, computational efforts may be an issue long-term because the genomic matrix needs to be created and inverted.

## Conclusions

Estimates of the additive genetic variance with pedigree or joint pedigree-genomic relationships are similar when the differences between the average diagonal and the average off-diagonal elements in **G **are similar to those in **A**. Adding the genomic information to **A **results in lower standard errors of additive variance estimates. Accuracies of EBV with the pedigree-genomic matrix are a function not only of the average of diagonal and off-diagonal elements of **G**, but also of the difference between these averages. The accuracy estimates may be inflated with non-normalized **G**. Matrix compatibility can be obtained by using observed allele frequencies and re-scaling the genomic relationship matrix to obtain average diagonal elements equal to 1. If allele frequencies in the base population are different from 0.5, rare alleles contribute more to the genetic resemblance between individuals than common alleles.

## Competing interests

The authors declare that they have no competing interests.

## Authors' contributions

SF performed data edition, statistical analysis and drafted the manuscript. IA developed scripts for genomic computations and helped in statistical analysis. IM provided core software, mentored statistical analysis and made substantial contributions for the results interpretation. All authors have been involved in drafting the manuscript, revising it critically and approved the final version.
